# Exploratory disease mapping: kriging the spatial risk function from regional count data

**DOI:** 10.1186/1476-072X-3-18

**Published:** 2004-08-26

**Authors:** Olaf Berke

**Affiliations:** 1 Department of Population Medicine, Ontario Veterinary College, University of Guelph, Guelph, Ontario, CANADA, N1G 2W1; 2 Department of Biometry, Epidemiology and Information Processing, School of Veterinary Medicine Hannover, Bünteweg 2, D-30559 Hannover, Germany

## Abstract

**Background:**

There is considerable interest in the literature on disease mapping to interpolate estimates of disease occurrence or risk of disease from a regional database onto a continuous surface. In addition to many interpolation techniques available the geostatistical method of kriging has been used but also criticised.

**Results:**

To circumvent these critics one may use kriging along with already smoothed regional estimates, where smoothing is based on empirical Bayes estimates, also known as shrinkage estimates. The empirical Bayes step has the advantage of shrinking the unstable and often extreme estimates to the global or local mean, and also has a stabilising effect on variance by borrowing strength, as well. Negative interpolates are prevented by choice of the appropriate kriging method. The proposed mapping method is applied to the North Carolina SIDS data example as well as to an example data set from veterinary epidemiology. The SIDS data are modelled without spatial trend. And spatial interpolation is based on ordinary kriging. The second example is included to demonstrate the method when the phenomenon under study exhibits a spatial trend and interpolation is based on universal kriging.

**Conclusion:**

Interpolation of the regional estimates overcomes the areal bias problem and the resulting isopleth maps are easier to read than choropleth maps. The empirical Bayesian estimate for smoothing is related to internal standardization in epidemiology. Therefore, the proposed concept is easily communicable to map users.

## Background

As with the analysis of any set of data, it is always good practice to begin by producing and inspecting graphs. A feel for the data can then be obtained and any outstanding features identified. In spatial epidemiology this is called disease mapping. Bithel [7], Diggle [14] and Lawson [25] provide recent reviews of disease mapping. Spatial epidemiology comprises at least three types of study focus [17,25]. These are (i) disease mapping, (ii) disease clustering and (iii) geographical correlation analysis but these distinctions are not strict. For example a disease map is also used for reporting the results of a geographical correlation study or to highlight areas of high or low disease incidence, i.e. cluster locations in a cluster study [4,14]. But in the following, disease mapping is considered as exploratory analysis used to get an impression of the geographical or spatial distribution of disease or the corresponding risk. For this the disease map should be based on smoothed estimates, clean of noise and adjusted for variation in 'at-risk' population [[24], p. 163]. The resulting disease map should provide insight into possible causes, effects and trends in the vast amount of data. This will provide an invaluable starting point for epidemiologic enquiry.

There are three basic types of disease maps corresponding to certain types of data. These are dot maps for point (or case-event) data, choropleth maps for regional data (also called lattice- or census-tract data), and lastly, isopleth maps for geostatistical data (also called point measurements) representing spatially continuous phenomena at a limited number of sampling locations. Spatial epidemiology is mainly concerned with the analysis of two types of data: case-event data and regional summary data, generally leading to dot maps and choropleth maps, respectively. However, epidemiology is also concerned with the identification of unknown risk factors, which may be part of the environment such as air pollution, radiation or magnetic fields. And such factors vary spatially continuously. Thus it is important to produce an isopleth map of disease occurrence or risk. Furthermore, as has been often pointed out in the literature [[24], p. 131], choropleth maps must be interpreted with caution, as the grey scale grouping is arbitrary and such a choice can affect interpretation, although choropleth maps imply a constant risk (incidence or prevalence) over regions with discontinuities at the border lines. Another point of criticism concerns the areal bias [12], as the varying shape and size of geographical regions make the patchy maps difficult to interpret. The visual impact of larger areas is higher and may dominate the map, leading to biased visual perception, whereas in human epidemiology it is the smaller, urban areas, and not the rural surroundings, that are of primarily interest due to population sizes. These objections may be circumvented by the use of continuous surface mappings, i.e. isopleth maps.

## Methods

### Review of interpolation methods

For spatial point case-control data Bithell [6] introduced a spatial interpolation method based on kernel density estimation. The resulting map is called the "spatial relative risk function". Lawson and Williams [28] and Kellsall and Diggle [20] proposed modifications of this procedure.

Regional data arise from summarising individual information for administrative regions such as census tracts. The basic model for the individual data, i.e. spatial point data, is the spatial Poisson process. In case of a rare disease, aggregation of the data over distinct regions results again in Poisson distributed data. For a more common disease the regional counts may be binomially distributed.

When spatial interpolation of regional data is the objective of the disease mapping study a grid of surface interpolant co-ordinates must be provided. Then a number of techniques can be used to automatically interpolate the data by use of deterministic methods or to predict the values statistically at the grid co-ordinates. Among the possible choices are kernel smoothing, splines, loess and running medians; see [19] for a discussion of these methods. Kernel smoothing has the advantage of preserving the positivity condition implied in rate data. Brillinger [8] used kernel-type smoothing in the context of birth rate data, and Müller, Stadtmüller and Tabnak [33] applied locally weighted least squares adapted for spatial aggregation to AIDS incidence maps for the San Francisco Area.

Another approach for the interpolation of regional data onto a continuous surface is the geostatistical prediction method of kriging. Some implementations of kriging have been proposed to obtain a risk surface [10,23,31,34,35,38]. Kelsall and Wakefield [21] have proposed a more complex geostatistical approach based on generalised linear modelling (GLIM) of regional data, which is similar to the work by Diggle, Tawn and Moyeed [15]. The relative merits of different interpolation approaches haves not so far been systematically investigated [[24], p. 131].

### Kriging the spatial risk function

The problems with the kriging method for generating isopleth maps of disease occurrence, i.e. the spatial risk function, are: (1) heterogeneous variances in the regional estimates and (2) the potential of negative interpolations. The first problem can be ameliorated by the use of empirical Bayes estimation to smooth the data prior to kriging. Appropriate geostatistical modelling can solve the second problem.

Let the study area be divided into *N *disjunctive sub regions indexed by *i *(*i = *1*,..., N*). Define *n*_*i *_as the size of the 'at-risk' population of the *i*-th region, and denote the number of cases by *m*_*i*_. The proportion *p*_*i *_= *m*_*i*_*/ n*_*i *_is the crude estimate for the parameter of interest *θ*_*i*_.

#### Smoothing by empirical Bayes estimation

Mapping raw estimates of disease occurrence can lead to spurious spatial features. To overcome this problem Cressie and Read [13] have explored the use of several variance stabilising transformations, but the results on the transformed scale are difficult to interpret. Furthermore, empirical Bayesian methods have been developed based on the idea of pooling information across regions. The resulting smoothed regional estimates have a variance stabilising side effect by borrowing strength from (local or global) neighbourhood information. The outline of the empirical Bayes approach for smoothing regional rates of rare diseases is based on the Poisson model. For the case of more common diseases Martuzzi and Elliott [30] adapted the approach to the Binomial model.

#### Poisson model for rare diseases

Clayton and Kaldor [11] proposed empirical Bayes or shrinkage estimation for smoothing regional data along with maximum likelihood estimation (MLE) of the unknown prior parameters. Marshall [29] modified this approach using method of moments estimators (MME) instead of MLE. The resulting estimates provide starting values for the iterative maximum likelihood procedure; or they could be used for exploratory mapping purposes, which is the main interest of this work.

Assume the cases in every region *i *are independently Poisson distributed with the unknown parameter *θ*_*i *_which has an unknown prior distribution associated with expectation E(*θ*_*i*_) = *π *and variance Var(*θ*_*i*_) = *φ*^2^. Then the totals of cases from the *i*-th region, e.g. *m*_*i *_(*i = *1*,..., N*), are distributed as follows

*m*_*i *_| *θ*_*i*_, *n*_*i *_~ Po(*n*_*i *_*θ*_*i*_)

*θ*_*i *_~ (*π*, *φ*^2^)

The MME of the unknown hyperparameters are  for the prior mean and  for the prior variance, where  denotes the mean regional 'at-risk' population and the summation is over the range of *i*. The empirical Bayesian estimates then becomes  with shrinkage weights .

#### Tendency of variance homogeneity

As stated above, the smoothing of the crude regional estimates *p*_*i *_has the side-effect of stabilising the variances for the regional empirical Bayes estimates, i.e. . Heuristically this is clear, because the empirical Bayes estimates are based on the whole sample information and not just on the individual regional sample, and thus are more stable. But this is difficult to prove, because this requires an analytical expression for the corresponding variance or an approximate variance estimate. Morris [32] proposed an approximate variance estimate for the empirical Bayes estimate in the Gaussian/Gaussian setting. However, an extension of this idea to the non-Gaussian setting is awkward [[9], p. 80].

Therefore, two facts are used to claim the conjecture that the empirical Bayes estimates of the regional estimates show up the tendency of variance homogeneity. First, it should be noted that Bayes estimates, i.e. , generally have a smaller associated variance than the corresponding frequentist estimates, i.e. *p*_*i *_[32]. This means the Bayesian smoothing generally results in reduced variances, thus reducing the absolute differences between regional variances, i.e. the variance heterogeneity. Secondly, by virtue of the shrinkage property of the empirical Bayes estimate, it follows that the variance will be shrunk back to the global variance in the case of small regional samples, which is responsible for a large part of the unstable estimates. Both points together make up what is called "borrowing strength from the ensemble" [32].

#### Geostatistical modelling

The geostatistical method of kriging is widely accepted for the purpose of spatial prediction, i.e. interpolation and (moderate) extrapolation. It is proposed here to predict the smoothed regional data onto a fine meshed regular grid of points for isopleth mapping purposes.

#### Spatial linear model

Kriging of spatial data, say *Z *= (*Z*_1_,..., *Z*_*N*_)' at sample sites *s*_*i*_, i = 1,..,*N*, takes place within the framework of the spatial linear model [1,12]

*Z *= *μ *+ *δ*,     E(*Z*) = *μ*,     *δ *~ Gau(0, Σ)

An integral part of this model is spatial correlation, which must be taken into account to draw valid scientific inferences. Here Σ is a variance-covariance matrix, spatially structured according to the position and direction between sampling sites. The basic assumption for spatial data is that near things are more related than distant things. This was neglected for smoothing of the regional epidemiological measures (prevalence or incidence) via empirical Bayes estimators.

#### Semivariogram

For geostatistical modelling, the structure of spatial variation will be estimated through the semivariogram , where *h *denotes the translation between any two arbitrary sites *s*_*i *_and *s*_*j *_within the study region. See [12,37] for diverse parameterised semivariogram models and estimation methods.

#### Kriging

In geostatistics kriging is used synonymously with optimal spatial prediction. However the optimality depends on the appropriateness of the spatial model. Cressie [12] gives a review on diverse kriging approaches. There are different models with respect to the knowledge and estimation of the spatial mean function, i.e. *μ*(*s*). Ordinary kriging is concerned with an unknown but constant mean function, i.e. *μ*(*s*) = *μ*. Furthermore, universal kriging is based on a polynomial trend surface model which is to be removed prior to estimation of the semivariogram from the residuals, i.e. *δ*(*s*). This technique may be the most widely-used in practice. An outlier-resistant alternative is median polish kriging. This method starts by the robust and non-parametric estimation of the non-constant mean surface via median polishing followed by robust semivariogram estimation. Berke [2] proposed a modification of median polish kriging for larger spatial data sets.

Generally, kriging surfaces are the sum of an estimate for the trend surface *μ*(*s*) plus the kriging prediction for the residual process *δ *(*s*), formally .

#### Kriging and smoothing

Kriging is sometimes termed a smoothing method. This is due to the fact that the predicted residuals  are in absolute not larger than the model residuals *δ*(*s*) = *Z*(*s*) - *μ*(*s*), i.e. the variability of the predictions  around the estimated mean surface  is smaller than the variability of the observations *Z*(*s*). When the semivariogram is modelled without nugget effect, i.e. without small-scale variability at spatial scales smaller than the observational scale, then kriging leads to direct interpolation at the sampling sites. In this case the prediction equals the observation at the sample sites and thus the predicted residuals are equal to the model residuals, i.e. . Predictions at any other sites have the tendency to shrink towards the value of the estimated trend surface at that place. On the other hand, when a semivariogram with nugget effect is appropriate, than the prediction tends to be closer to the mean surface, which gives smaller residuals , i.e. a smoother prediction surface . Thus the prediction of invalid values for risk or for other epidemiologic measures, as has been criticised in the past, is a consequence of inappropriate spatial modelling. This could only happen when universal kriging is based on an estimated trend surface model , which exceeds the range of valid values.

## Results

The mapping technique proposed to generate isopleth risk maps from regional count data is now applied to two example data sets. Example 1 is based on the SIDS mortality rates in North Carolina that show no spatial trend. Example 2 is based on spatial trend contaminated data of tapeworm infections among red foxes in Lower Saxony.

### Example 1: sudden infant death syndrome (SIDS) in North Carolina

This by now classical spatial data set on SIDS mortality rates in North Carolina from 1974 to 1984 has been analysed by many researchers. Cressie [12] gives an introduction to the problem, earlier references and results on data modelling, mapping and cluster detection. More recently Kulldorff [22] applied the spatial scan test to detect clusters of disease and Lawson and Clark [27] applied a spatial mixture modelling approach to map the standardized mortality ratio (SMR) for the period 1974 – 1978.

**Figure 1:**
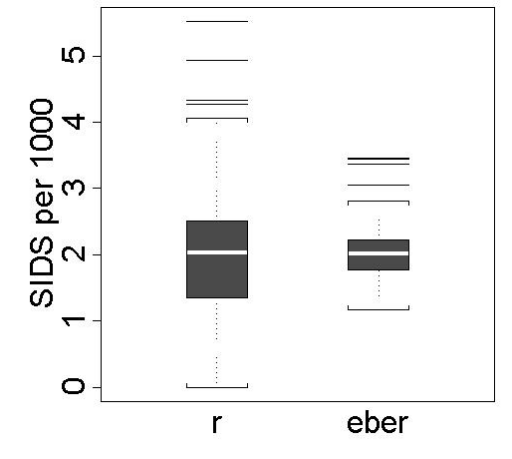
**Comparison of raw and Bayesian smoothed SIDS mortality rates. **Parallel box plots for the raw annual SIDS mortality rate per 1000 live births (*r*) and the corresponding shrinkage estimates or empirical Bayesian estimated rates (*eber*) from 100 counties of North Carolina, 1974–1984.

For the years 1974 to 1984 the number of live births ranges from 567 to 52345 over North Carolina's 100 counties. The total number of reported SIDS cases is 1503 out of 753354 live births, which results in an annual mortality rate of approximately 2 per 1000 live births. The mean of the counties boundary files coordinates in longitude and latitude were used here as the geographic coordinates for the regions centres. Figure 1 shows the shrinkage effect by using parallel box plots for the raw rates and the empirically Bayesian smoothed rates under the Poisson model for rare phenomena.

**Figure 2:**
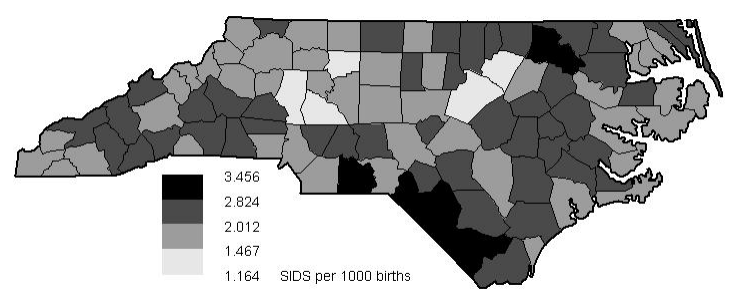
**Choropleth map of Bayesian smoothed SIDS mortality rates of North Carolina. **Choropleth map of the empirical Bayesian smoothed mortalities per 1000 live births from 100 counties of North Carolina, 1974–1984. Cut points of the grey scale shading are the 5%, 50% and 95% quantiles of the empirical distribution.

The smoothed rates are more appropriate for disease mapping than the raw rates and hence used here for choropleth mapping in Figure 2. Cut points of the grey scale shading are the 5%, 50% and 95% quantiles of the empirical distribution. These are used to highlight the upper and lower five percent of the distribution of the Bayesian smoothed mortality rates and to distinguish between higher and lower values. The smoothed rates vary from about 1.2 to 3.5 cases per 1000 live births. Visual map perception reveals some potential high and low risk areas in the north and southwest but no striking spatial trend pattern.

**Figure 3:**
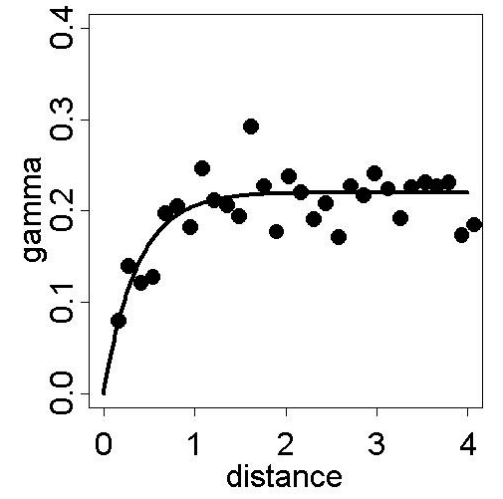
**Empirical semivariogram from smoothed SIDS mortality rates and fitted exponential model. **Exponential model (sill = 0.22, range = 0.37) fitted by weighted least squares (WLSE) to the robust empirical semivariogram from empirical Bayesian smoothed annual SIDS mortality rates per 1000 live births from 100 counties of North Carolina, 1974–1984.

Geostatistical prediction, i.e. kriging of the empirical Bayesian smoothed rates, is based on appropriate modelling of the data. Here, the constant mean assumption for the spatial mean surface is chosen and justified by visual inspection of the empirical semivariogram which levels out and reaches a sill. The spatial dependence structure is modelled by an isotropic exponential semivariogram without nugget effect, which is fitted by weighted least squares estimation to the robustly estimated empirical semivariogram; see [12] for technical details. The result of this procedure is summarised in Figure 3. The close fitting of the empirical semivariogram to the model indicates appropriate model choice for the dependence structure as well as for the constant mean surface. See [1] for diagnostic methods and its applications in geostatistical modelling.

**Figure 4:**
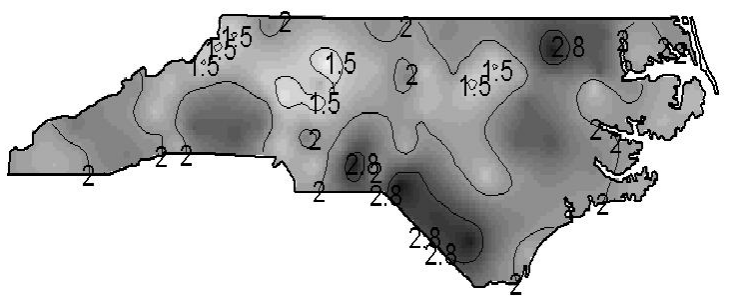
**Isopleth map from kriging the smoothed SIDS mortality rates of North Carolina. **Isopleth map based on kriging predictions of the empirical Bayesian smoothed annual SIDS mortality rates per 1000 live births from 100 counties of North Carolina, 1974–1984.

Due to the constant mean assumption ordinary kriging is the appropriate spatial prediction method and without nugget effect this leads to direct interpolation of the data at the counties centres and to smoothed values shrunken towards the global mean for the rest of the study area. The resulting isopleth map or risk surface map is given in Figure 4. Now the grey scale shading is almost continuous, i.e. the patchy nature of Figure 2 is replaced by a surface. Additional isolines are drawn for the same cut points as for the grey scale shadings in Figure 2 (i.e. at the 5%, 50% and 95% level of the empirical distribution of the empirical Bayesian estimates) and will be useful to support map interpretation and comparison. The risk surface map may be more useful to identify potential environmental risk factors than the choropleth map.

**Figure 5:**
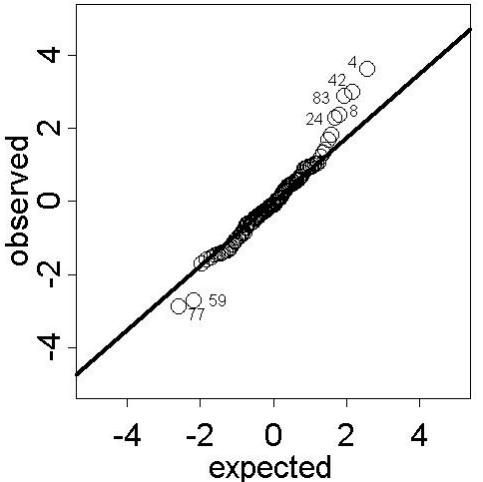
**Normal probability plot of cross-validation residuals from kriging smoothed SIDS mortality rates. **Normal probability plot of the cross-validation residuals from kriging the empirical Bayesian smoothed regional SIDS mortality rates. Observations 4, 42, 83, 8 and 24 as well as 77 and 59 may be outliers with respect to the spatial model.

Cross-validation can be used to explore the predictive performance of the kriging model. The cross-validation residuals (weighted with respect to the spatial dependence structure given by the semivariogram) should be approximately Gaussian distributed. Normal probability plots can be used to disclose grossly model inadequacies [[12], p. 498] as well as for outlier identification. Figure 5 shows the normal probability plot of the cross-validation residuals based on the refitted model with the outliers removed.

Aside from some positive and negative extreme values the general appearance of the residual process is Gaussian. The potential outliers are regions with steep gradients in risk and hence part of disease clusters (or their surroundings) which were previously identified [22] and are located in the north and south-west of North Carolina. Of course, disease clusters are of interest and the proposed modelling approach reveals their existence and points to the respective high risk areas. This is in line with the gross aims of exploratory disease mapping.

### Example 2: tapeworm infections in red foxes in Lower Saxony

*Echinococcus multilocularis *(E.m.) is a tapeworm occurring in the northern hemisphere, including endemic regions in central Europe, most of northern and central Eurasia and parts of North America. In central Europe the red fox is the main definitive host with rodents such as mice or muskrats serving as intermediate hosts [16]. The parasite E.m. causes the zoonosis alveolar echinococcosis (A.E.), which has a potentially high fatality rate. Recent studies reflect an alarmingly wide geographic range of the parasite in foxes, with average prevalences varying up to 60% for central Europe. However, the spatial distribution of E.m. in foxes is complex and insufficiently known. There are indications of emerging risk factors for human A.E. such as increasing parasite prevalences in red foxes, growing fox population and progressive spread of foxes to cities.

The federal state of Lower Saxony is part of northern Germany and contains the federal city-state of Bremen as an enclave. During the period from 1991 to 1997, 5365 red foxes were sampled in Lower Saxony and examined for infections with E.m.. The data are given in Table 1[3].

**Table 1:** Regions and statistics for the fox tape worm example. The 43 investigated regions in Lower Saxony with their co-ordinates (x, y), the number of red foxes tested (n) and found positive for *E. multilocularis *(m), the raw period prevalence in % (PP) and the empirical Bayes smoothed period prevalence in % (BPP). The regions no. 5, 8, 13, 14 and 15 form a previously identified disease cluster [4].

**Regions**				**Statistics**			
	
**Nr.**	**Name**	**x**	**y**	**n**	**m**	**PP**	**BPP**
1	Braunschweig	7,57	-4,30	25	1	4	6
2	Salzgitter & Wolfenbüttel	7,47	-6,07	115	9	8	8
3	Wolfsburg	9,33	-2,83	22	6	27	23
4	Gifhorn	8,15	-1,37	158	13	8	8
5	Göttingen	3,44	-12,92	157	84	54	51
6	Goslar	6,68	-8,81	152	20	13	13
7	Helmstedt	10,11	-4,30	66	7	11	10
8	Northeim	2,86	-10,28	186	96	52	49
9	Osterode/Harz	6,68	-11,07	94	23	24	23
10	Peine	5,80	-4,11	115	10	9	9
11	Hannover	2,17	-2,64	327	41	13	12
12	Diepholz	-4,39	0,88	143	10	7	7
13	Hameln-Pyrmont	-0,17	-6,36	99	41	41	39
14	Hildesheim	3,74	-6,36	202	60	30	29
15	Holzminden	1,19	-8,62	60	16	27	24
16	Nienburg/Weser	-1,74	-0,58	325	20	6	6
17	Schaumburg	-1,25	-4,11	90	12	13	13
18	Celle	4,81	0,59	255	8	3	3
19	Cuxhaven	-3,51	10,69	73	14	19	18
20	Harburg	3,64	7,06	285	17	6	6
21	Lüchow-Dannenberg	11,68	3,92	225	20	9	9
22	Lüneburg	7,37	6,37	278	22	8	8
23	Qsterholz-Scharmbeck	-3,80	6,57	32	8	25	22
24	Rothenburg/Wümme	-0,17	6,47	114	6	5	5
25	Soltau-Fallingbostel	2,46	2,84	137	8	6	6
26	Stade	0,41	10,00	84	5	6	6
27	Uelzen	7,76	3,53	214	14	7	6
28	Verden	-1,45	3,44	107	11	10	10
29	Delmenhorst	-4,88	4,31	8	1	13	12
30	Emden	-14,74	8,19	4	0	0	9
31	Oldenburg	-6,84	4,12	103	15	15	14
32	Osnabrück	-9,19	-3,12	290	21	7	7
33	Wilhelmshaven	-8,55	10,30	10	0	0	6
34	Ammerland	-8,99	6,28	76	4	5	6
35	Aurich	-13,11	9,42	111	15	14	13
36	Cloppenburg	-9,78	2,84	110	8	7	7
37	Emsland	-13,60	1,07	242	21	9	8
38	Friesland	-9,10	9,32	15	1	7	9
39	Bentheim	-15,95	-1,46	36	1	3	4
40	Leer	-12,42	6,67	39	2	5	6
41	Vechta	-8,12	0,20	83	2	2	3
42	Wesermarsch	-6,54	7,55	45	9	20	18
43	Wittmund	-10,76	10,10	53	4	8	8
Sum				5365	706		
Median (in %)						8,2	9,0

**Figure 6:**
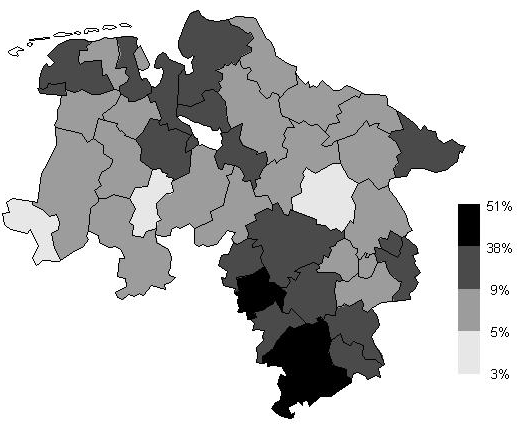
**Choropleth map of Bayesian smoothed prevalences in Lower Saxony. **Choropleth map of the empirical Bayesian smoothed period prevalences from 43 regions in Lower Saxony. Cut points of the grey scale shading are the 5%, 50% and 95% quantiles of the empirical distribution.

Figure 6 is a choropleth map of the empirical Bayes smoothed period prevalences from 43 regions in the study area. The cut points of the grey scale shading are again the 5%, 50% and 95% quantiles of the empirical distribution or the smoothed data. The period prevalences range from 3% to 51% with the median at 9%.

The map indicates high period prevalences in the south and north of Lower Saxony. Extraordinarily high prevalences were observed in the southern regions, which indicate the presence of a positive disease cluster that is identified by use of the spatial scan statistic [4].

**Figure 7:**
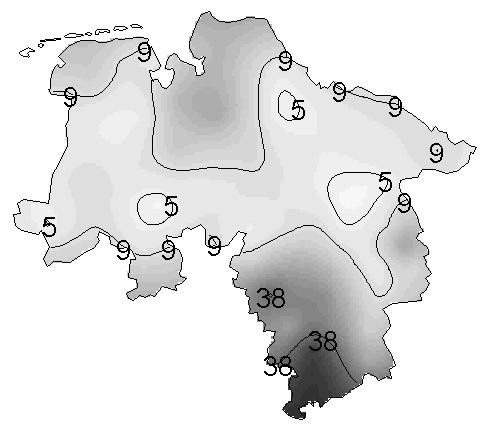
**Isopleth map from kriging the smoothed prevalences in Lower Saxony. **Isopleth map with overlaid isolines of the kriging interpolated choropleth map in Figure 6. Isolines are at the 5%, 50% and 95% quantiles of the empirical distribution of the empirical Bayesian smoothed regional period prevalences.

Figure 7 shows the corresponding isopleth map resulting from universal kriging, with overlaid isolines. The isopleth map gives an impression of gradual changes instead of jumps at the regional borders. Furthermore, the missing region of Bremen in the central north of Lower Saxony has also been supplied with predicted values.

**Figure 8:**
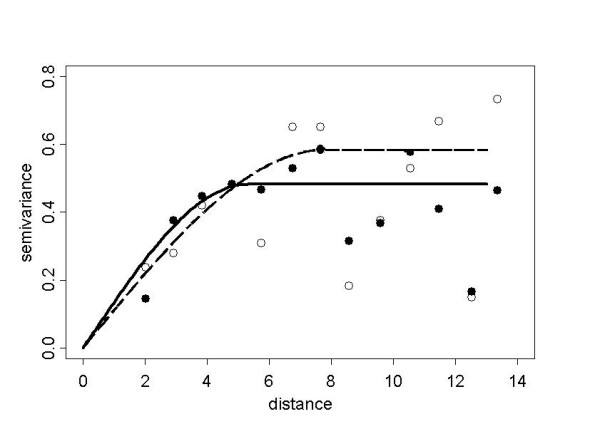
**Empirical semivariograms and fitted exponential models from detrended and trend-contaminated smoothed prevalences. **Robust empirical semivariograms of the detrended (black dot) and trend-contaminated data (circle) along with the WLSE fitted spherical models for the detrended (solid line) and the trend-contaminated (dashed line) data with observations 3 and 13 removed.

Figure 6 points out the presence of heterogeneity in the spatial mean. Here, universal kriging is based on the assumption that the spatial mean function of the data could be represented by an incomplete quadratic polynomial in the spatial co-ordinates. Other potential explanatory variables are not at hand. Let *s *= (*x*, *y*)' denote an arbitrary location in the study region, where *x *and *y *are the co-ordinates to the east and north. By inspection of the variogram cloud [12] based on trend residuals, two observations (Sites 3 and 13) were identified as outliers and removed from the structure analysis. In the second step of an iterated modelling approach then the trend polynomial fitted by ordinary least squares (OLSE) is given by *μ*(*s*) = *β*_0 _+ *β*_1_*y *+ *β*_2_*y*^2^, with , , . Instead of OLSE one could use iterated WLSE, but iteration may lead to biased estimates. The residuals of the trend surface fit to the empirical Bayes smoothed regional period prevalences were then used to model a spherical semivariogram by weighted least squares estimation of the robustly estimated empirical semivariogram. Figure 8 shows the empirical semivariogram as well as the fitted model for both, the detrended and trend contaminated data (sill = 0.0039, range = 5.29 and sill = 0.0047, range = 7.83 respectively), to indicate the benefit from the trend model as measured by the 20% decrease of the sill value.

**Figure 9:**
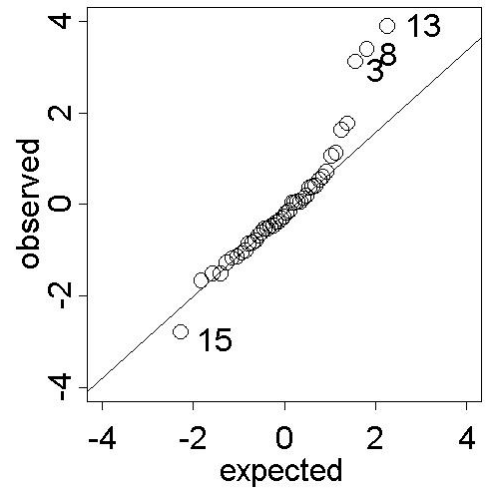
**Normal probability plot of cross-validation residuals from kriging smoothed prevalences. **Normal probability plot of the cross-validation residuals from kriging the empirical Bayesian smoothed regional period prevalences. Observations 3, 8 and 13 may be spatial outliers with respect to the model based on the data set with observations 3 and 15 removed. Regions 8 and 13 are nearest neighbours of region 15, all three of which are part of a positive cluster.

The normal probability plot of the kriging cross-validation residuals in Figure 9 draws attention to the presence of three or four outliers (Observations 3, 8 and 13 and possibly 15). Observation 3 and 15 were previously identified as extremes and excluded from the analysis. Regions 8 and 13 are the nearest neighbours of Region 15. All three are part of a positive spatial cluster [4], a spatial structure that could not be captured by the spatial linear model used for universal kriging. However, the general appearance of the data is Gaussian. This in turn justifies the appropriateness of the modelling and prediction approach based on the empirical Bayesian smoothed regional data.

**Figure 10:**
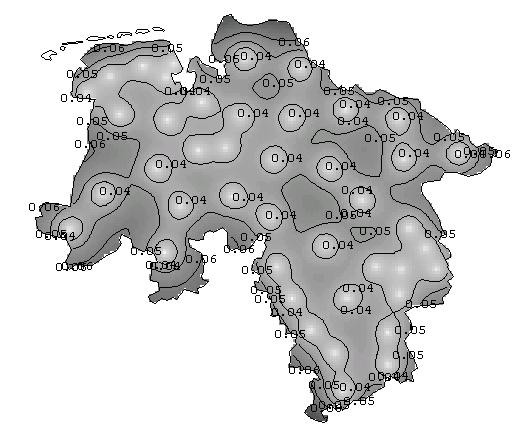
**Error map. **Error map based on the universal kriging standard errors.

Geostatistical modelling of the empirical Bayesian smoothed regional prevalences allows the calculation of an error map representing the kriging standard errors. The darker the error map in Figure 10 the larger are the kriging standard errors, which range up from 0 to 0.06.

## Summary and discussion

In this paper two sophisticated statistical methods were combined to solve an open problem in disease mapping. Mapping regional data using choropleth maps holds on to several problems that are overcome by isopleth mapping. To interpolate the regional, i.e. spatially discrete information, the geostatistical method of kriging is used here. Kriging requires variance homogeneous data. However, regional risk estimates are generally based on varying sample sizes and consequently turn up spatially varying standard errors. Therefore the spatial risk estimates are smoothed using linear empirical Bayes estimation. By borrowing-strength-from-the-ensemble, the impact of outliers is reduced and standard errors are stabilized over space. This cannot achieve variance homogeneity entirely nor could the tendency be shown analytically. Bootstrapping methods for spatially dependent data [18] promise an empirical justification and will be a source for future research.

The purpose of exploratory disease mapping is to provide insight, as opposed to precise estimates of location, spread or trends [19]. Emphasise shall be on easy and intuitive statistical mapping methods. The method proposed here for exploratory disease mapping is on one hand based on empirical Bayesian estimation for smoothing which is related to internal standardization in epidemiology [[9], p. 260] and could thus be viewed as a natural choice for adjusting the data for spatially varying variances. On the other hand, the kriging predictions are weighted moving averages, where weights are chosen with respect to the spatial autocorrelation structure exhibited by the sample data. This concept is easily communicable to map users. Only monochrome colours or shades of grey should be used along with isopleth mapping [36], as has been done here in order to avoid the natural preference of the human eye for bright colours and in deference to colour-blind map users.

Kriging is known to be a smoothing method and it may be argued that the proposed mapping method results in double or over-smoothing. But here kriging is based on a semivariogram model without nugget effect, which is known to be a direct interpolation method [12]. Therefore the risk map shows the Bayesian smoothed regional risks in the respective regional centres.

The proposed disease mapping approach is a two-step procedure. The corresponding error map (Figure 10), however, neglects the error from the first step, i.e. the smoothing step. This is in line with error maps used along with similar sandwich predictors obtained via median polish kriging [3,12]. Thus the error map is useful to investigate the predictive performance of the kriging step but less useful for analytical inferences.

The empirical Bayes method as described here does not take into account spatial autocorrelation, but a modification to overcome this lack has been proposed [29]. The method described thus far is called global smoothing, because the Bayes estimates are shrunk towards the global mean of all regions. The modification consists of using the local mean based on neighbouring regions instead of shrinking the estimates, which is called local smoothing. In any case, a simulation-based evaluation of a wide range of estimation methods [26] shows that the global smoother performs better overall than the local smoother. (This result may be related to the fact, that also regions with higher or lower population density cluster into urbanized regions and rural surroundings.) Only the full Bayesian approach to hierarchical modelling of regional disease data [5] outperforms the global smoother. But this modelling approach is certainly not suited for exploratory work.

## Conclusions

Interpolation of the regional disease risk estimates overcomes the areal bias and related problems of choropleth disease mapping. Consequently, isopleth maps are easier to read and interpret than choropleth maps. The geostatistical method of kriging is appropriate for this task when based on linear empirical Bayesian smoothed data. Unlike non-parametric and mathematical interpolation methods, the spatial model underlying the exploratory spatial risk map offers ways of interpretation.

Finally, the proposed concept for exploratory spatial risk mapping is easily communicable to map users. The Bayesian smoothing estimator is related to internal standardization in epidemiology. Also kriging can be viewed as weighted moving averaging.
